# Chromatin Interaction Responds to Breast Muscle Development and Intramuscular Fat Deposition Between Chinese Indigenous Chicken and Fast-Growing Broiler

**DOI:** 10.3389/fcell.2021.782268

**Published:** 2021-11-29

**Authors:** Weihua Tian, Zhang Wang, Dandan Wang, Yihao Zhi, Jiajia Dong, Ruirui Jiang, Ruili Han, Zhuanjian Li, Xiangtao Kang, Hong Li, Xiaojun Liu

**Affiliations:** ^1^ College of Animal Science and Technology, Henan Agricultural University, Zhengzhou, China; ^2^ Henan Innovative Engineering Research Center of Poultry Germplasm Resource, Zhengzhou, China; ^3^ International Joint Research Laboratory for Poultry Breeding of Henan, Zhengzhou, China

**Keywords:** Hi-C, A/B compartment, TAD, muscle development, IMF deposition, chicken

## Abstract

Skeletal muscle development and intramuscular fat (IMF) content, which positively contribute to meat production and quality, are regulated by precisely orchestrated processes. However, changes in three-dimensional chromatin structure and interaction, a newly emerged mediator of gene expression, during the skeletal muscle development and IMF deposition have remained unclear. In the present study, we analyzed the differences in muscle development and IMF content between one-day-old commercial Arbor Acres broiler (AA) and Chinese indigenous Lushi blue-shelled-egg chicken (LS) and performed Hi-C analysis on their breast muscles. Our results indicated that significantly higher IMF content, however remarkably lower muscle fiber diameter was detected in breast muscle of LS chicken compared to that of AA broiler. The chromatin intra-interaction was prior to inter-interaction in both AA and LS chicken, and chromatin inter-interaction was heavily focused on the small and gene-rich chromosomes. For genomic compartmentalization, no significant difference in the number of B type compartments was found, but AA had more A type compartments versus LS. The A/B compartment switching of AA versus LS showed more A to B switching than B to A switching. There were no significant differences in the average sizes and distributions of topologically associating domains (TAD). Additionally, approximately 50% of TAD boundaries were overlapping. The reforming and disappearing events of TAD boundaries were identified between AA and LS chicken breast muscles. Among these, the *HMGCR* gene was located in the TAD-boundary regions in AA broilers, but in TAD-interior regions in LS chickens, and the *IGF2BP3* gene was located in the AA-unique TAD boundaries. Both *HMGCR* and *IGF2BP3* genes exhibited increased mRNA expression in one-day-old AA broiler breast muscles. It was demonstrated that the *IGF2BP3* and *HMGCR* genes regulated by TAD boundary sliding were potential biomarkers for chicken breast muscle development and IMF deposition. Our data not only provide a valuable understanding of higher-order chromatin dynamics during muscle development and lipid accumulation but also reveal new insights into the regulatory mechanisms of muscle development and IMF deposition in chicken.

## Introduction

Chicken is often regarded as one of the most desirable meats worldwide due to its proteins, polyunsaturated fatty acids (PUFA), calcium, phosphorus, and iron content, as well as its potential medicinal function and low cost (Soriano-Santos, 2010). In recent decades, the genetic selection for body weight, growth rate, and feed conversion rate of broiler chickens has contributed to a prominent increase in production efficiency, such as meat yield; however, there has also been a parallel decrease in meat quality. The synchronized improvement of both meat yield and quality has become a hot topic in molecular breeding in chicken. Skeletal muscle constitutes the largest proportion and most valuable component of meat yield, and its development and composition are of extreme importance in meat production efficiency and quality, particularly breast muscle in poultry.

Skeletal muscle is mainly composed of muscle fibers derived from the first formation of primary muscle fibers at 3.5 embryos old and the subsequent formation of secondary muscle fibers at 15 embryos old until hatching during the embryonic period in chicken (Bandman and Rosser, 2000; Picard et al., 2002). Myoblasts undergo first proliferation, followed by the fusion to form myotubes, then final differentiation into muscle fibers. Muscle growth and development go through two stages, an increase in the number of proliferative myofibers (termed hyperplasia) from the embryonic stage to early growth stage, and an increase in the size of myofibers (termed hypertrophy) during late growth stages. It has been demonstrated that the total number and morphological structure of myofibers are fixed before hatching or birth in poultry (Smith, 1963). Muscle development after birth mainly profits from growing muscle fiber diameter and length, which involves complex regulatory networks and signaling pathways. Compared to slow-growing native chicken, fast-growing commercial broilers showed a significant increase in diameter, cross-sectional area, and density of breast muscle fibers at hatching. It has been proposed that heavier chicks could have enhanced satellite cell proliferation and earlier differentiation. The increased activity of muscle satellite cells in the late stage of the embryo could make breast muscle development faster (Velleman et al., 2000; Sklan et al., 2003).

It was reported that myocytes and adipocytes could affect each other during development, and meat-producing animals that have increasing muscularity displayed a reduced intramuscular fat (IMF) development (Hocquette et al., 2010). IMF refers to the lipid between muscle fibers, mainly distributed in epimysium, perimysium, and endomysium. IMF content serves as a deeply predominant factor positively underlying meat quality, including flavor, tenderness, and juiciness, since IMF is particularly rich in phospholipids, an important precursor substance carrying unsaturated fatty acid. IMF content depends upon the hyperplasia (number) and hypertrophy (size) of adipocytes in muscle. Generally, the number of adipocytes, which are mainly determined by genetic factors, is stationary during embryonic and early development stages. The size of adipocyte refers to the ability of lipid droplet accumulation, which is determined by lipid hydrolysis and traffic of both endogenous exogenous triglyceride in the blood, uptake across cytomembrane, and metabolism including fatty acids esterification, oxidation, and lipolysis in the cytoplasm (Qiu et al., 2017). IMF deposition is mainly regulated by genetic and nutritional factors with medium to high heritability. Increasing research on the difference in meat quality among chicken breeds has emphasized the comparison of fast-growing commercial broilers and slow-growing native chicken breeds, indicating that the latter has superiority and priority in meat quality and palatability (Leenstra, 1986; Chen et al., 2005; Li et al., 2006; Hocquette et al., 2010; Chen et al., 2016). It has been established that the IMF level at the beginning of the growth period was a likely key determinant of the final IMF level in cattle, indicating such significance of IMF accumulation in the early period. The chicken IMF content showed a significant increase from day 56 (fast-growth age), day 98 (marketing age), and day 140 (first egg age), but reached a peak at hatching in which the protein expression profile of breast muscles was especially varied from the other ages (Liu et al., 2016). It was similar to duck, which manifests a maximum lipid level and the relative area occupied by adipocytes on cross-sections of breast muscle (Chartrin et al., 2007). Another case in point also showed that the major period of IMF deposition in chicken was from embryo day 17 to day 1 (Liu R. et al., 2017). This evidence supports the idea that more attention should be paid to IMF deposition at the early period, especially the hatching.

It has been confirmed that no genetic conflict occurs between meat quality and meat production, implying that selection is valuable to synchronously improve them in broilers (Le Bihan-Duval et al., 2008). Both skeletal muscle development and IMF deposition are sophisticated and precisely orchestrated processes mediated by a network of regulatory factors. Although several studies have highlighted the roles of coding and noncoding genes in skeletal muscle development and IMF deposition, their precise molecular mechanism has largely remained elusive, especially three-dimensional chromatin structure and interaction, which have been gradually characterized as critical new regulators and have been recognized as an emerging mediator for triggering gene expression (de Laat and Duboule, 2013; Dekker and Mirny, 2016).

High-through chromosome conformation capture (Hi-C) technique has recently been developed to study the genome-wide spatial chromatin organization and obtain genome-wide and high-resolution chromatin intra- (*cis*) and inter- (*trans*) interaction to elucidate gene regulation mechanisms, consequently phenotype formation (Belton et al., 2012; Stadhouders et al., 2012; Liu et al., 2019). Chromatin is characterized with high conformation by hierarchical structural units organized into following chromosome territory (CT), compartments including transcriptionally active gene-rich compartment A (open euchromatin) and transcriptionally inactive gene-desert compartment B (closed heterochromatin), regions of more localized interaction known as topologically associating domain (TAD) as well as chromatin loop in the nucleus (Gibcus and Dekker, 2013). It has been largely acknowledged that compartment B has a higher interaction intensity than compartment A, and A/B compartment switch (A to B and B to A) directly aids in the activation or inhibition of gene expression. TADs represent local contact-enriched areas within the compartment, which correspond to relatively isolated packing units of gene expression regulation with the spatial proximity of genes and their regulatory elements located in a long linear region (Dixon et al., 2012). Between TADs, there are boundaries, called TAD boundaries. TAD boundaries maintain TAD independence to characterize by self-association and insulation, which respectively promotes the gene promoter interaction with regulatory elements within TAD or avoids that across TAD borders, and play a crucial role in stabilizing genome structure and regulating gene expression (de Laat and Duboule, 2013; Ibrahim and Mundlos, 2020). However, the disappearing and remodeling of TAD boundaries may lead to abnormal intergenic interactions and altered gene expression levels (Dixon et al., 2015). Chromatin loops facilitate long-range interactions of the promoter with enhancer and silencer. They can affect gene regulation to a certain extent while dynamic formation and the disappearance of the loop structure occur (Stadhouders et al., 2012).

Lushi blue-shelled-egg chicken (LS) is a Chinese indigenous breed with the strengths of crude feed tolerance, strong adaptability, high disease resistance, as well as favorable meat quality. However, this breed has a low growth rate, feed conversion ratio, and meat production. Arbor Acres broiler (AA) is a specialized fast-growing commercial broiler, characterized as having fast weight gain, a high feed conversion ratio, and massive meat production, especially breast muscle.

To determine the regulation mechanisms underlying genome organization and genome-wide chromatin interactions during skeletal muscle development and IMF deposition, we comprehensively determined the chromatin interaction, A/B compartment switch, TAD distribution, and potential genes and signaling pathways, and unveiled gene regulation mediated by chromatin interaction at the chromosome scale using Hi-C in the breast muscles from commercial broiler AA and Chinese native Lushi chicken. Our data not only reveal a connection between three-dimensional chromatin interaction with muscle development and IMF deposition but also provide a valuable genomic resource for new insights into understanding the molecular mechanisms of skeletal muscle development and IMF deposition in chicken.

## Materials and Methods

### Ethics Statement

All birds used in this study were obtained from the Animal Center of Henan Agricultural University. The procedures of all animal experiments were approved by the Institutional Animal Care and Use Committee of Henan Agricultural University (Permit Number: 11-0085). All efforts were made to humanely slaughter the birds and consider animal welfare.

### Experimental Animals and Sample Preparation

All the experimental chickens were raised in the same feeding environment with free access to water and feed. Breast muscles tissues were collected from 10-embryonic-old (E10), 14-embryonic-old (E14), 18-embryonic-old (E18), 1-day-old (1D), 3-week-old (3W), and 5-week-old (5W) AA and LS chicken (*n* = 6), respectively. Subsequently, they were immediately frozen in liquid nitrogen and stored at −80°C. A total of 30 5-week-old male Lushi and AA chickens (15 individuals for each breed) were selected to measure body weight, shank length, shank circumference, sternum length, body oblique length, as well as depth, width, and angle of breast muscle. Then, blood was collected from the wing vein into a 5 ml coagulation tube, underwent an incubation for 2 h, and centrifuged at 3,000 r/min for 10 min at room temperature for serum collection. According to the manufacturer’s specifications (Huili, Changchun, China), the serum biochemical indexes including alanine transaminase (ALT), aspartate transaminase (AST), glucose (GLU), lactate dehydrogenase (LDH), triglyceride (TG), total cholesterol (T-CHO), high density lipoprotein (HDL-c), low density lipoprotein (LDL-c) and very low-density lipoprotein (VLDL-c) were tested. After being humanely slaughtered, the breast muscle was harvested and infiltrated in 4% paraformaldehyde for measuring muscle fiber diameter by hematoxylin-eosin (HE) staining, intramuscular fat content by oil red O staining, and muscle TG, T-CHO content by Tissue TG and T-CHO ELISA kits (Sinobestbio, Shanghai, China) following manufacturer’s introductions. A total of 40 one-day-old male Lushi and AA chickens (20 individuals for each breed) were used to measure the birth weight. Then, based on the high uniformity of birth weight, three one-day-old chickens each breed were selected to collect breast muscle samples and equivalently mixed into one composite sample, respectively, for Hi-C analysis.

### HE Staining

The breast muscle fiber diameter was measured by hematoxylin-eosin staining as described by Carvalho (de Carvalho and Taboga, 1996). The paraffin sections of breast muscle were immobilized with 10% paraformaldehyde (Servicebio, Wuhan, China) for 5 min, and were washed with distilled water, then stained with hematoxylin for 15 min. Following 1% hydrochloric acid-ethanol differentiation for several seconds, the paraffin sections were subsequently stained with 0.5% eosin for 3 min and then went through gradient alcohol dehydration for 2 min and vitrification by dimethylbenzene for 2 min. The cell nucleus was stained blue, and the cytoplasm was stained pink. All reactions were performed in triplicate.

### Oil Red O Staining

The breast muscle samples (*n* = 3 each group) were sectioned with a freezing microtome. The frozen sections of breast muscle were immobilized with 10% paraformaldehyde (Servicebio, Wuhan, China) for 30 min, washed three times with 1× phosphate buffer saline (PBS) (Gibco, Gaithersburg, MD, United States), and then incubated with oil red O solution (Servicebio, Wuhan, China) for 15 min. Finally, the cell nuclei of breast muscle tissue were re-stained with hematoxylin (Servicebio, Wuhan, China) for 5 min. The lipid droplet was stained red, and the cell nucleus was stained blue.

### Preparation and Sequencing of Hi-C Libraries

The collected fresh muscle samples were cut into pieces with 2–3 mm diameter on the ice and washed in PBS (Gibco, United States). The samples were centrifugated at 4°C, 2,000 × g for 10 min and were resuspended with serum-free Dulbecco’s Modified Eagle’s medium (DMEM) (Gibco, Gaithersburg, MD, United States). The resuspended cells were DNA-protein cross-linked by a final 2% concentration of formaldehyde, incubated at room temperature for 10 min with soft shock at an interval of 2 min. The cross-linked samples were then supplemented with a final concertation of 0.2 M glycine and incubated at room temperature for 5 min, followed by 15 min on the ice to completely terminate cross-linking, and collected by centrifugation at 4°C, 2,000 × g for 10 min. Total DNA, gelatinin protein residues, and chromatin integrity were used to evaluate the crosslinking effect using the proteinase K DNA extraction method, in concert with agarose gel electrophoresis. The qualified samples were stored at −80°C for further use.

Hi-C experiment was performed as previously reported with minor modifications (Lieberman-Aiden et al., 2009). Briefly, chromatin digestion and isolation were performed by restriction enzyme MboI at 37°C, and the resulting sticky ends were filled in with nucleotides and introduced biotinylated nucleotides at ligation junctions. After that DNA fragment ends were ligated with T4 DNA ligase, purified with a ranging size from 300 to 500 bp, and sheared. Biotinylated junctions were isolated with streptavidin beads, then sequencing connectors were added to form joint products to construct the Hi-C library with an Illumina TruSeq DNA Sample Prep Kit according to the manufacturer’s instructions. Hi-C libraries were sequenced on an Illumina Novaseq 6000 platform configured for 150 bp paired-end reads. The Hi-C experiment was performed by Annoroad Gene Technology Co., Ltd. (Beijing, China).

### Hi-C Data Analysis

Clean Hi-C data were generated by filtering out low-quality reads (>15% of bases with Q scores ≤ 19%), reads containing over 5% poly-N, and adapters from Hi-C raw data using Trimmomatic (Bolger et al., 2014). Clean reads were mapped to Gallus gallus 5.0 reference genome with a two-step approach embedded in HiC-Pro V2.7.8 software. The unique mapped paired-end reads were generated by filtering out the unmapped paired-end reads, paired-end reads with singleton and multi mapped paired-end reads from clean reads. Then the reads with single side mapping, self-circle ligation, dangling ends, dumped pairs and duplicated valid pairs from PCR artifacts were discarded. Only valid read pairs with unique mapped paired-ends involving two kinds of restriction fragments were used to build the contact maps. The valid paired-end reads were divided into intrachromosomal interactions (*cis*) reads and inter-chromosomal interactions (*trans*) reads, where *cis* rate and *trans* rate respectively referred to the proportion of *cis* and *trans* interaction paired-end reads in the valid paired-end reads. The raw contact maps were normalized with a sparse-based implementation of the iterative correction method by HiC-Pro and visualized by HiTC (Servant et al., 2012). Normalized interchromosomal interaction matrix was obtained from observed/expected number of contacts between all pairs of whole chromosomes to evaluate the spatial proximity of chromosomes. We performed an overall Hi-C subtraction of the Z score matrices (AA minus LS) with genomic distance using a modified LOWESS method, by which the weighted-average and weighted-standard deviation for every genomic distance were calculated to consequently normalize for genomic distance signal bias (Sanyal et al., 2012).

### A/B Compartment Analysis

The A/B compartments were estimated by eigenvector analysis of the genome contact matrix after normalization using the observed/expected method (Lieberman-Aiden et al., 2009). Pearson correlation matrix responding to the correlation between each of two loci on the chromosome and the whole genome interaction were employed to dramatically sharpen the plaid pattern. In the normalized interaction matrix heat map, blue indicates a lower observed interaction frequency than expected interaction frequency, on the contrary to red. The correlation coefficient ranged from −1 to 1, and the color transited from blue to red. According to principal component analysis (PCA) analysis with dimensionality reduction of the genome contact correlation coefficient matrix, the chromatin region could be distinctly divided into two parts according to the first eigenvector, which was called the A/B compartment. The positive values and negative values in the first eigenvector respectively, determined the A and B compartments.

### TAD Calling and TAD Boundary Analysis

To determine TADs and their boundaries, we proceeded with the previously reported insulation score method (Crane et al., 2015). Briefly, an “insulation score” that reflects the aggregate of interactions occurring across each interval was assigned to genomic intervals along the chromosome to quantify TADs. Of which, the sum of interaction strength of the local chromosome regions was calculated, after smooth transformation, these valleys/minima were interpreted as TAD boundaries or areas of high local insulation, wherein that the minima of insulation profile in total local interaction located at the TAD boundary. It involves two steps, first, the interaction strength value of each bin was calculated by summing its interaction strength in the 10 kb binned Hi-C data (the number of unique mapped read pairs to these regions), subsequently, the interaction strength of each bin served as a value to draw its fluctuation curve on the chromosome to define the boundary.

### Prediction and Functional Analysis of miRNAs Target Genes

The miRanda (http://www.microrna.org/microrna/home.do) and TargetScan (http://www.targetscan.org/vert_72/) were employed to predict the potential target genes of miRNA. Gene Ontology (GO) and Kyoto Encyclopedia of Genes and Genomes (KEGG) pathway enrichment analysis of genes was conducted using DAVID (http://david.abcc.ncifcrf.gov/) with a cut-off criterion of *p* value ≤ 0.05.

### RNA Extraction and Complementary DNA (cDNA) Synthesis

Total RNA was extracted from the breast muscle tissues of E10, E14, E18, 1D, 3W, and 5W AA and LS chickens (*n* = 6 each group) using TRIzol^®^ reagents (Invitrogen, Carlsbad, CA) according to the manufacturer’s protocol. The RNA integrity was detected using 1% TAE agarose gel electrophoresis and Agilent Bioanalyzer 2100 system (Agilent Technologies, CA, United States). The concentration, as well as purity assessed by OD260nm/280 nm and OD260nm/230nm, of RNA samples, were measured using NanoPhotometer^®^ spectrophotometer (IMPLEN, CA, United States). The RNA samples with the ratios of 28S/18S band brightness more than 1.5, as well as 1.8–2.0 of OD260 nm/280 nm and 2.0–2.3 of OD260 nm/230 nm were reversely transcribed into cDNA using PrimerScriptTM RT reagent kit (Takara, Kyoto, Japan) following the manufacturer’s instructions.

### Quantitative Real-Time PCR (qRT-PCR)

To detect mRNA expression profiles, SYBR green-based qRT-PCRs were performed in triplicate on a LightCycler^®^96 Real-Time PCR system (Roche Applied Science, United States) in a 10 μl reaction volume containing 5 μl 2 × SYBR^®^ Premix Ex Taq™ II (Takara, Kyoto, Japan), 3 μl RNase-free water, 0.5 μl each of forward and reverse primers (10 μM), and 1 μl cDNA (about 300 ng). The qRT-PCR amplification procedure comprised an initial denaturation at 95°C for 5 min; 40 cycles of denaturation at 95°C for 30 s, annealing at optimum temperature for 30 s and extension at 72°C for 30 s; followed by a final extension at 72°C for 10 min. The housekeeping gene *GAPDH* was served as an internal control to normalize the relative gene expression. The mRNA relative expression was calculated using the 2^−∆∆Ct^ method. The primers were designed via NCBI Primer-BLAST (https://www.ncbi.nlm.nih.gov/tools/primer-blast/) and synthesized by Sangon Biotech (Shanghai, China). The qRT-PCR primer sequences are represented as follows: *IGF2BP3*-F: GCC​TTG​GCA​GTT​GGA​GCT​AT, *IGF2BP3*-R: AGC​TTG​GCA​TCT​GGT​CCT​TC; *HMGCR*-F: GGT​GTC​CTT​GTC​CGG​GGA​G, *HMGCR-R*: GGC​CAT​GCA​TTC​GAA​AAA​GC; *GAPDH*-F: AGA​ACA​TCA​TCC​CAG​CGT, *GAPDH*-R: AGC​CTT​CAC​TAC​CCT​CTT​G.

### Statistical Analysis

All data were presented as mean ± standard deviation (SD), and the statistical significance between the two experimental groups was evaluated by *t*-test for comparisons using SPSS 23.0 (IBM, Chicago, IL, United States). The *p value < 0.05 was considered statistically significant, and **p value < 0.01 was considered extremely significant. Graphics were produced via GraphPad Prism 8 (GraphPad Software, San Diego, CA, United States).

## Results

### The Differences of Live Weight, Body Size Indexes, Serum Biochemical Indexes, Breast Muscle Fiber Diameter, and IMF Content Between AA Broiler and Lushi Chicken

**FIGURE 1:**
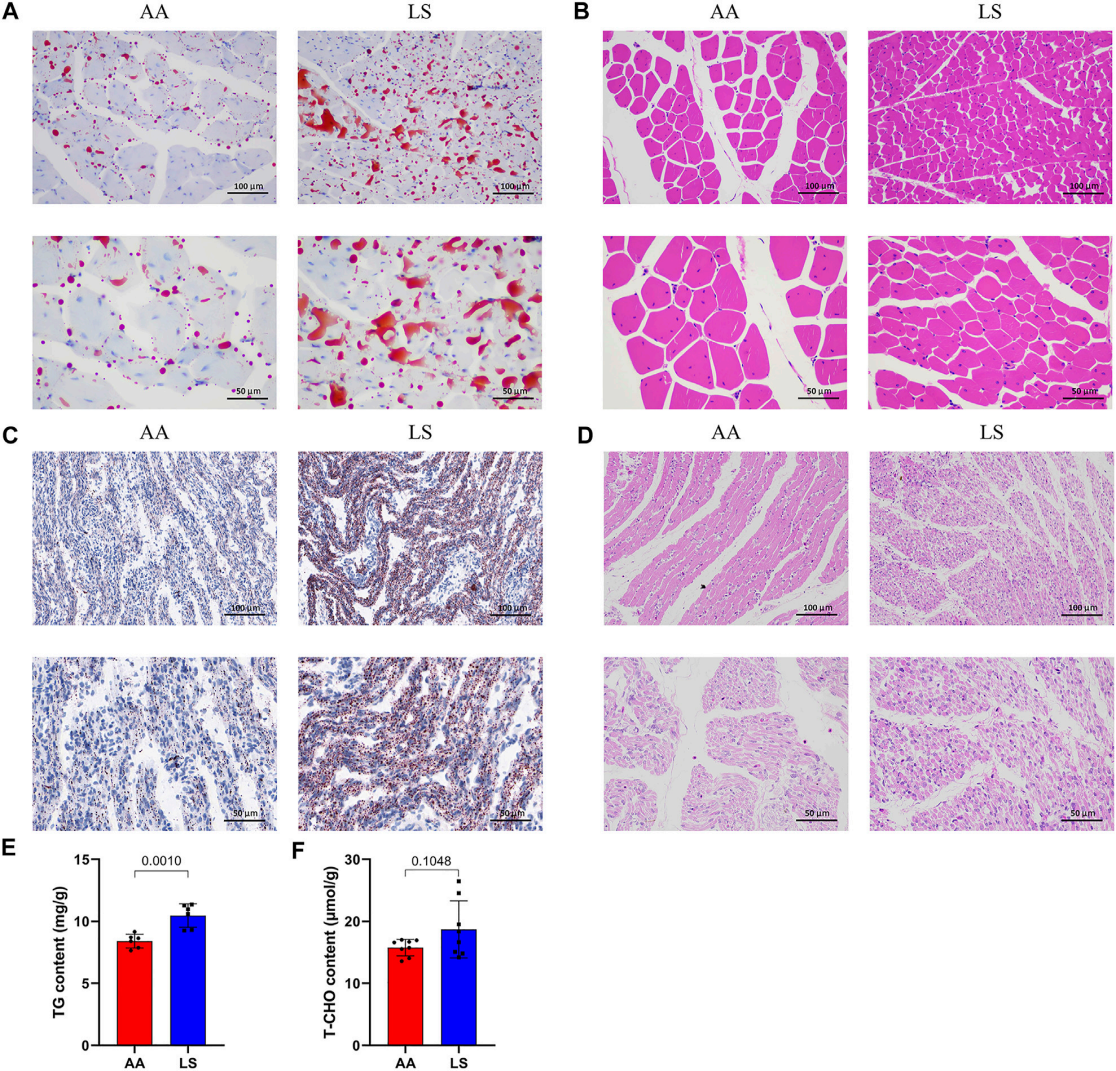
IMF deposition and fiber diameter in LS chicken breast muscle compared with AA broiler. **(A)** The difference in lipid droplet accumulation in the breast muscles of 5-week-old AA broiler and LS chicken as determined by oil red O staining. **(B)** The difference in breast muscle fiber diameter in the breast muscles of 5-week-old AA broiler and LS chicken determined by HE staining. **(C)** The difference in lipid droplet accumulation in the breast muscles of one-day-old AA broiler and LS chicken determined by oil red O staining. **(D)** The difference in breast muscle fiber diameter in the breast muscles of one-day-old AA broiler and LS chicken determined by HE staining. Scale bar: 100 μm (20X), 50 μm (40X). **(E,F)** The concentrations of TG and T-CHO, respectively, in the breast muscle of AA broiler and LS chicken (*n* = 8 for each group).

Compared with Lushi chicken, AA broiler is characterized by extremely significant higher live weight (*p* = 3.35E-22) and body size indexes at 5 weeks old including shank length (*p* = 3.68E-19), shank circumference (*p* = 1.47E-14), sternal length (*p* = 2.02E-22), body length (*p* = 8.87E-27), chest depth (*p* = 3.23E-23), chest width (*p* = 9.19E-23) and chest angle (*p* = 3.31E-20) (Table 1). There was no significant difference in the serum levels of ALT (*p* = 0.581), AST (*p* = 0.367), GLU (*p* = 0.083), LDH (*p* = 0.823), and TG (*p* = 0.112) between 5-week-old AA broiler and Lushi chicken, although serum TG exhibited an increased trend in LS chicken. Of note, significantly higher serum T-CHO (*p* = 0.005), HDL-c (*p* = 0.005), LDL-c (*p* = 0.001), and VLDL-c (*p* = 0.047) were found in LS chicken versus AA broiler (Table 1). The remarkably increased IMF content of pectorals (Figure 1A), but remarkably decreased breast muscle fiber diameter (Figure 1B) were observed in Lushi chicken in comparison with AA broiler at 5 weeks old. Simultaneously, the conspicuously superior IMF content was uncovered in one-day-old LS chicken compared to the AA broiler (Figure 1C), but the latter exhibited a prominent breast muscle fiber diameter in pectorals (Figure 1D). Moreover, significantly increased TG level was observed in the muscle of one-day-old LS chicken compared to AA (*p* = 0.0010) (Figure 1E), and there was no significant difference of muscle T-CHO between them (*p* = 0.1048) (Figure 1F).

**TABLE 1:** The difference of body sizes and serum biochemical indexes between 5-week-old AA broiler and Lushi chicken.

Indexes	AA	LS	*p* Value
Live weight (kg)	2.28 ± 0.260^A^	0.36 ± 0.024^B^	3.35E-22
Shank length (mm)	78.68 ± 3.18^A^	53.75 ± 2.188^B^	3.68E-19
Shank circumference (mm)	10.31 ± 0.790^A^	5.62 ± 0.330^B^	1.47E-14
Sternal length (cm)	14.79 ± 0.716^A^	6.60 ± 0.442^B^	2.02E-22
Body length (cm)	25.03 ± 0.887^A^	11.03 ± 0.937^B^	8.87E-27
Chest depth (mm)	67.71 ± 2.889^A^	41.67 ± 1.497^B^	3.23E-23
Chest width (mm)	53.80 ± 2.445^A^	28.13 ± 2.182^B^	9.19E-23
Chest Angle (°)	100.67 ± 3.27^A^	71.93 ± 2.19^B^	3.31E-20
ALT (U/L)	26.393 ± 15.262	21.935 ± 1.114	0.581
AST (U/L)	302.105 ± 120.624	243.241 ± 5.691	0.367
GLU (mmol/L)	11.507 ± 0.472	12.997 ± 1.200	0.083
LDH (U/L)	658.435 ± 86.635	682.662 ± 178.447	0.823
TG (mmol/L)	0.746 ± 0.067	0.831 ± 0.061	0.112
T-CHO (mmol/L)	3.949 ± 0.364^B^	5.722 ± 0.633^A^	0.005
HDL-c (mmol/L)	1.114 ± 0.062^b^	1.691 ± 0.2619^a^	0.005
LDL-c (mmol/L)	1.880 ± 0.075^B^	2.535 ± 0.157^A^	0.001
VLDL-c (mmol/L)	0.956 ± 0.253^b^	1.497 ± 0.353^a^	0.047

Note: ALT, alanine transaminase; AST, aspartate transaminase; GLU, glucose; LDH, lactate dehydrogenase; TG, triglyceride; T-CHO, total cholesterol; HDL-c, high density lipoprotein; LDL-c, low density lipoprotein; VLDL-c, very low-density lipoprotein. Different lowercase letters indicate significant difference (*p* < 0.05); different uppercase letters indicate extremely significant difference (*p* < 0.01). The same below.

### Characterization of Hi-C Data From AA and LS Breast Muscles

As shown in Table 2, 648,484,686 and 635,659,872 raw reads were produced from AA and LS, respectively. After filtration, clean reads totaling AA 622,163,611 (92.31%) and LS 609,409,215 (92.55%) were respectively, obtained with a Phred quality value >30 occupying 95.86 and 96.25%. Subsequently, AA 416,641,076 (66.97%) and LS 413,930,207 (67.92%) unique mapped paired-end reads from clean reads were mapped to chicken genome. After removal of dangling end paired-end reads, self-circle paired-end reads, and dumped paired-end reads, 377,935,675 (90.71%) and 314,219,395 (75.91%) unique mapped paired-end reads involving two kinds of restriction fragments, termed interaction paired-end reads, were respectively, obtained in AA and LS HiC libraries. After a final removal of reads with duplicate sequences from PCR amplification artifacts, a total of 173,384,380 (41.61% in AA unique mapped paired-end reads) and 170,953,363 (41.3% in LS unique mapped paired-end reads) valid paired-end reads were respectively, recognized for further analysis.

**TABLE 2:** Characteristics of the Hi-C reads from two chicken pectorales libraries.

Sample	AA	LS
Raw Paired-end Reads	648,484,686	635,659,872
Clean Paired-end Reads	622,163,611	609,409,215
Raw Q30 Bases Rate (%)	92.31	92.55
Clean Q30 Bases Rate (%)	95.86	96.25
Unique Mapped Paired-end Reads	416,641,076	413,930,207
Unique Mapped Ratio (%)	66.97	67.92
Dangling End Paired-end Reads	11,255,901	74,878,974
Dangling End Rate (%)	2.7	18.09
Self Circle Paired-end Reads	886,074	1,084,246
Self Circle Rate (%)	0.21	0.26
Dumped Paired-end Reads	26,563,426	23,747,592
Dumped Rate (%)	6.38	5.74
Interaction Paired-end Reads	377,935,675	314,219,395
Interaction Rate (%)	90.71	75.91
Valid Paired-end Reads	173,384,380	170,953,363
Valid Rate (%)	41.61	41.3
*Cis* Paired-end Reads	153,821,754	144,606,957
*Cis* Rate (%)	89.98	83.4
*Trans* Paired Reads	17,131,609	28,777,423
*Trans* Rate (%)	10.02	16.6
Theoretical Fragments	2,366,597	2,366,597
Reality Fragments	2,301,426	2,294,819
Reality Fragments Ratio (%)	97.25	96.97

### Comprehensive Maps of Chromatin Interaction in the Breast Muscles of AA and Lushi Chickens

**FIGURE 2:**
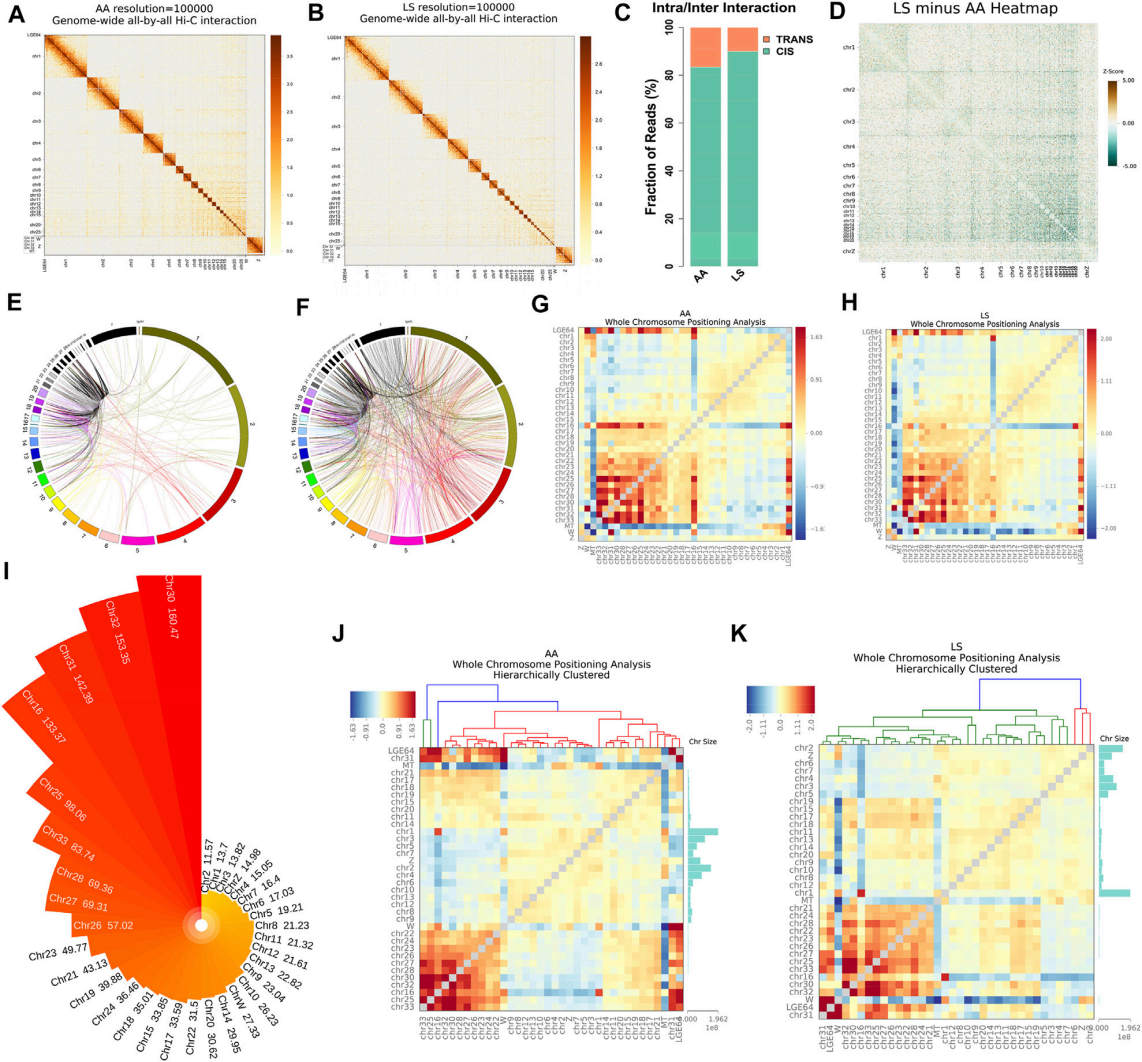
Chromatin *cis*/*trans* interaction analysis of AA and LS chicken breast muscle using Hi-C. **(A,B)** Genome-wide all-by-all Hi-C interaction heatmap of breast muscles in AA broiler and LS chicken, respectively. **(C)** Genome-wide *cis*/*trans* interaction percentage of AA and LS chicken breast muscles. **(D)** Genome-wide heatmap of significant differential interactions between AA and LS chicken breast muscles. Chromosomes are stacked from top left to bottom right from chr1 through chr20 and chrZ. The red color represents LS-enriched interactions and the green color represents AA-enriched interactions, the white regions are the insignificantly changed interacting regions between the two breeds. **(E,F)** Circle plot from 1,000 bin pairs with the strongest *trans*-interaction in AA and LS chicken breast muscles, respectively. **(G,H)** Standardized *trans*-interaction using observed/expected number of contacts between all pairs of whole chromosomes in AA and LS chicken breast muscles, respectively. **(I)** Gene density of each chromosome in chicken. **(J,K)** Hierarchical clustering analysis of whole chromosome positioning between chromosomes of AA and LS chicken breast muscles, respectively.

Whole genome-wide intra- (*cis*) and inter- (*trans*) interaction data of AA and LS chicken breast muscles were visualized as chromosome versus chromosome heat maps, where darker colors represent more frequent interaction events (Figures 2A,B). Of which, 89.98% of AA interaction data and 83.4% of LS interaction data were found as *cis* interaction, 10.02 and 16.6% as *trans* interaction (Figure 2C). Then, to evaluate whether the chromosomes clustering was altered between AA and LS chicken breast muscles, we compared the chr1-20 and chrZ genome-wide interaction differences and draw LS minus AA genome-wide heatmap of significant differential interactions. It was demonstrated that compared with AA, LS was equipped with significant increases in genome-wide interaction (Figure 2D). In order to intuitively show the *trans*-interaction, 1,000 bin pairs with the strongest interaction among chromosomes were used to draw circle plots. The circle plots showed that there was frequent interaction between the small, gene-rich, and high gene density chromosomes in AA and LS chicken breast muscles. Compared to LS, AA was equipped with the relatively repressed interaction frequency between large chromosomes and small chromosomes, in parallel with more frequent interaction between the large chromosomes (Figures 2E,F). Moreover, we standardized the interaction between each chromosome using the observed/expected number of contacts between all pairs of whole chromosomes to get a standardized chromosome interaction matrix in AA and LS chicken breast muscle, where the higher interaction between two chromosomes represents closer physical proximity (Figures 2G,H). As such, AA broiler breast muscle was characterized by more frequent interaction between small, gene-rich chromosomes, especially Chr25-33, in parallel with LS showing a similar trend except for the weaker interaction between Chr16 and Chr17-33. Considering that chromosome size made a difference in chromosomal interaction, we sorted all chromosomes subjected to gene density and conducted hierarchical clustering to exhibit the correlation more intuitively between chromosome size and the intensity of chromosomal interaction according to chromosomal interaction values. It was revealed that Chr30 with a size of 0.025 Mb possessed the highest gene density, following Chr32, Chr31, Chr16, Chr25, Chr33, Chr28, Chr27, Chr26, Chr23, Chr21 raging in size from 0.04 to 7 Mb (Figure 2I). Interestingly, hierarchical clustering analysis of whole chromosome positioning indicated that the chromosome *trans*-interaction was heavily concentrated on the small and gene-rich chromosomes in AA and LS chicken breast muscle (Figures 2J,K).

### Identification and Switching Characterization of A/B Compartments in the Breast Muscles

**FIGURE 3:**
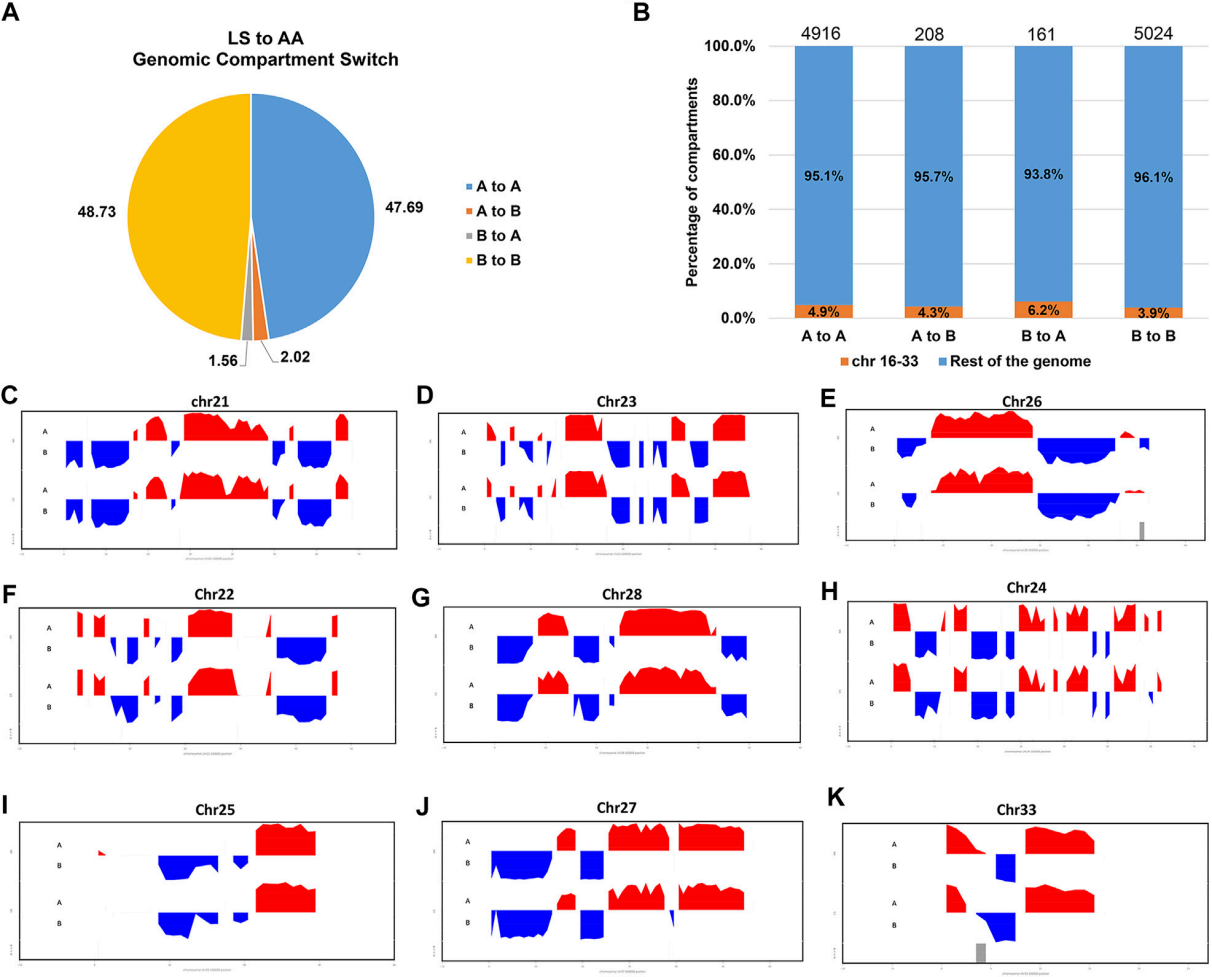
Genomic compartmentalization of AA and LS chicken breast muscles. **(A)** The genomic compartment changes between AA and LS chicken genomes from breast muscles. “A” and “B” mean the open and closed compartments, respectively. “A to A” means open compartments in both chickens, “B to B” represents closed compartments in both chickens, “A to B” means the compartments that are open in AA but closed in LS, and “B to A” means the compartments that are closed in AA and open in LS. **(B)** The percentage of compartments that have switched (A to B or B to A) or been stable (A to A or B to B) between AA and LS chicken breast muscle genomes for chr16 through chr33 (orange) and the rest of the genome (blue). **(C–K)** First principal component of chr21, chr23, chr26, chr22, chr28, chr24, chr25, chr27 and chr33, respectively. The upward red irregular rectangles represent open A-type compartmentalization and the downward blue irregular rectangles represent closed B-type compartmentalization.

To verify whether there are any differences in the compartmentalization between the AA and LS genomes from breast muscles, we compared the A/B compartments throughout the whole genome at 100 kb resolution. There was no significant difference in the number of B type compartments between the two breeds, except more A type compartments in AA versus LS (Table 3). AA and LS chicken breast muscles shared 47.69% stable A to A compartments and 48.73% stable B to B compartments, where 2.02% of all compartments constituted a switching in genomic compartmentalization from A-type in AA to B-type in LS, referring to A to B switching, and 1.56% vice versa (Figure 3A; Supplementary Table S1). To further explore whether small and gene-rich chromosomes dominatingly contributed to the A/B compartment switching, the compartment distribution of Chr16-33 and the rest of the genomes were analyzed. A distinct increase of stable A compartments and compartments switching from A-type in AA to B-type in LS on these chromosomes were found (Figure 3B). The small, gene-rich chromosomes possessed relatively abundant A compartment and poor B compartment in proportion versus large, gene-poor chromosomes, in either LS or AA chicken genomes from breast muscle (Supplementary Figure S1). We emphasized the A/B compartment switching on Chr21-33 and observed that there was no compartment switching on Chr21, on which both AA and LS merely constituted 1.45% B to A compartment switching, together with 52.17% stable A compartments and 46.38% stable B compartments (Figure 3C; Supplementary Figure S2A). As for Chr23 and Chr26, there were powerful A-type compartments with a significant increase of compartment switching from B-type in LS to A-type in AA, in which B to A compartment switching occupied 6.90%, A to B occupied 1.72% on Chr23, as well as respectively, 7.27 and 1.82% on Chr26 (Figures 3D,E; Supplementary Figures S2B,C). On Chr22, AA and LS genomes displayed a similar distribution for the A and B compartments, which had approximately equivalent percentages, and had the same proportion of A/B compartments switching (1.89% for each of A to B and B to A switching) (Figure 3F; Supplementary Figure S2D). In terms of Chr28, both AA and LS merely constituted stable A compartments and 44% stable B compartments (Figure 3G; Supplementary Figure S2E). Only A to B compartment switching was respectively, detected on Chr24 (3.17%), Chr25 (2.86%), Chr27 (1.75%), and Chr33 (11.76%) in the breast muscles of the two kinds of chickens (Figures 3H–K; Supplementary Figures S2F,G–I).

**TABLE 3:** The numbers of A/B compartment in genomes of AA and LS chicken breast muscles.

Chr	AA		LS	
	A-compartment	B-compartment	A-compartment	B-compartment
chr1	917 (0.32)	1990 (0.68)	919 (0.32)	1990 (0.68)
chr2	727 (0.33)	1,480 (0.67)	712 (0.32)	1,480 (0.68)
chr3	545 (0.33)	1,094 (0.67)	542 (0.33)	1,094 (0.67)
chr4	428 (0.31)	940 (0.69)	431 (0.31)	940 (0.69)
chr5	294 (0.34)	580 (0.66)	297 (0.34)	580 (0.66)
chr6	180 (0.35)	332 (0.65)	172 (0.34)	332 (0.66)
chr7	192 (0.37)	334 (0.63)	195 (0.37)	334 (0.63)
chr8	146 (0.33)	292 (0.67)	141 (0.33)	292 (0.67)
chr9	122 (0.36)	220 (0.64)	118 (0.35)	220 (0.65)
chr10	97 (0.33)	196 (0.67)	93 (0.32)	196 (0.68)
chr11	85 (0.28)	220 (0.72)	84 (0.28)	220 (0.72)
chr12	97 (0.32)	204 (0.68)	93 (0.31)	204 (0.69)
chr13	88 (0.33)	180 (0.67)	85 (0.32)	180 (0.68)
chr14	88 (0.41)	126 (0.59)	86 (0.41)	126 (0.59)
chr15	74 (0.42)	104 (0.58)	75 (0.42)	104 (0.58)
chr17	61 (0.42)	84 (0.58)	62 (0.42)	84 (0.58)
chr18	57 (0.35)	104 (0.65)	56 (0.35)	104 (0.65)
chr19	56 (0.41)	82 (0.59)	59 (0.42)	82 (0.58)
chr20	72 (0.34)	138 (0.66)	68 (0.33)	138 (0.67)
chr21	36 (0.36)	64 (0.64)	37 (0.37)	64 (0.63)
chr22	21 (0.33)	42 (0.67)	21 (0.33)	42 (0.67)
chr23	31 (0.40)	46 (0.60)	34 (0.43)	46 (0.58)
chr24	38 (0.43)	50 (0.57)	36 (0.42)	50 (0.58)
chr25	12 (0.32)	26 (0.68)	11 (0.30)	26 (0.70)
chr26	26 (0.36)	46 (0.64)	29 (0.39)	46 (0.61)
chr27	35 (0.44)	44 (0.56)	34 (0.44)	44 (0.56)
chr28	28 (0.39)	44 (0.61)	28 (0.39)	44 (0.61)
chr33	13 (0.62)	8 (0.38)	11 (0.58)	8 (0.42)
Z	409 (0.38)	678 (0.62)	399 (0.37)	680 (0.63)
Sum	4,975 (0.34)	9,748 (0.66)	4,928 (0.34)	9,750 (0.66)

Note: Chr represents chromosome. The numbers in parentheses denotes the ration of A or B compartment on chromosomes.

### Functional Annotation and Signaling Pathway Enrichment of Genes Located on the A/B Compartment Switch Region in the Breast Muscles

**FIGURE 4:**
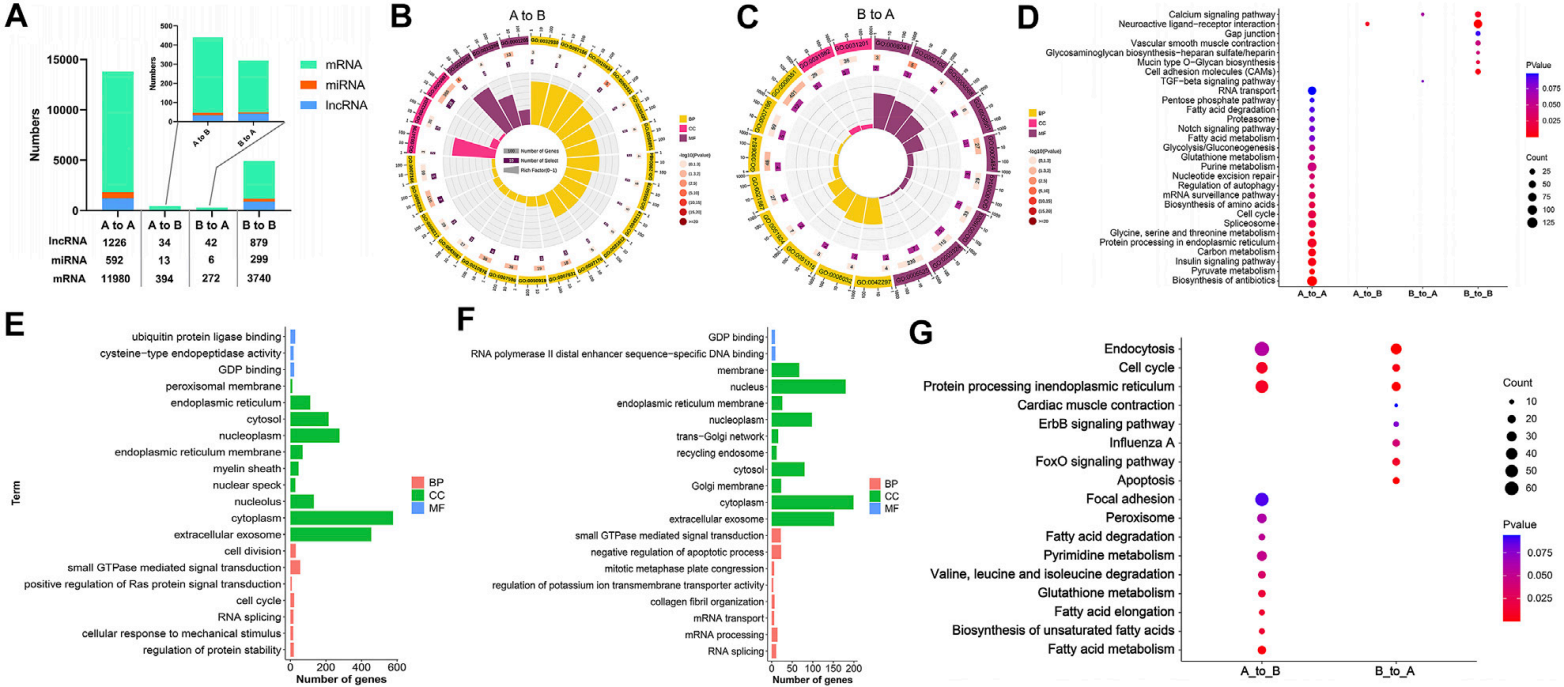
Functional annotation and signaling pathway enrichment of genes located on the A/B compartment switch region between AA and LS chicken breast muscles. **(A)** Distribution of lncRNA, miRNA, and mRNA in A to A, A to B, B to A, B to B compartments regions between AA and LS chicken genomes from breast muscles. **(B)** GO enrichment analysis of mRNAs within A to B compartment switching regions. **(C)** GO enrichment analysis of mRNAs within B to A compartment switching regions. **(D)** KEGG pathways analysis of mRNAs in A to A, A to B, B to A, B to B compartments regions. **(E)** GO enrichment analysis of potential targeting mRNAs of miRNAs in A to B compartment regions. **(F)** GO enrichment analysis of potential targeting mRNAs of miRNAs in B to A compartment regions. **(G)** KEGG pathways analysis of potential targeting mRNAs of miRNAs in A to B and B to A compartments regions.

A total of 1,226 long chain non-coding RNAs (lncRNAs), 592 microRNAs (miRNAs), and 11,980 mRNAs, were respectively, authenticated in the stable A compartments at the whole genome scale. Among those, 34 lncRNAs, 13 miRNAs, and 394 mRNAs in A to B compartment switching region, 42 lncRNAs, 6 miRNAs, and 272 mRNAs located in B to A compartment switching region, as well as 879 lncRNAs, 299 miRNAs, and 3,740 mRNAs in stable B compartments (Figure 4A; Supplementary Table S2).

To better understand the correlation of genomic compartmentalization with regulating muscle development and IMF deposition between AA and LS, we performed the functional annotation and signaling pathway enrichment analysis of genes located in the A/B switching region in their breast muscles. A total of 25 clusters based on the GO functional annotation of mRNAs in the A to B compartment switching region were validated. There were nine significant terms including negative regulation of platelet aggregation (*p* = 0.0030), cell proliferation (*p* = 0.0132), blood coagulation (*p* = 0.0253), positive regulation of neuron projection development (*p* = 0.0253), feeding behavior (*p* = 0.0395), negative chemotaxis (*p* = 0.0436), an integral component of the plasma membrane (*p* = 0.0123), thrombin receptor activity (*p* = 0.0019), and transcriptional activator activity, RNA polymerase II distal enhancer sequence-specific binding (*p* = 0.0224) (Figure 4B; Supplementary Table S3). Moreover, 19 clusters based on the GO functional annotation of mRNAs in B to A compartment switching region were validated, including five significant terms: cellular calcium ion homeostasis (*p* = 0.0334), dystroglycan binding (*p* = 0.0016), peptidyl-dipeptidase activity (*p* = 0.0382), SNAP receptor activity (*p* = 0.0469) as well as transcription, DNA-templated (*p* = 0.0351) (Figure 4C; Supplementary Table S3). Moreover, the top 20 GO terms from mRNAs in stable A and B compartments according to *p* value were respectively, selected and showed that the majority of terms focused on cellular components, following molecular function and biological process least in both stable A and B compartments (Supplementary Figures S3A,B and Supplementary Table S3).

To identify the crucial signaling pathways that participated in muscle development and IMF deposition between AA and LS, we mapped these genes respectively, from stable and switched compartments to KEGG orthologs. The genes on A to B compartment switching region were significantly enriched in the neuroactive ligand-receptor interaction pathway (*p* = 0.0129). Genes in the B to A compartment switching region were enriched in the calcium signaling pathway (*p* = 0.0632) and TGF-β signaling pathway (*p* = 0.0761). Concerning mRNAs on stable A compartment, they were significantly enriched in the biosynthesis of antibiotics (*p* = 2.15E-05), pyruvate metabolism (*p* = 0.0015), insulin signaling pathway (*p* = 0.0052), carbon metabolism (*p* = 0.0055), protein processing in the endoplasmic reticulum (*p* = 0.0095), spliceosome (*p* = 0.0228), cell cycle (*p* = 0.0236), mRNA surveillance pathway (*p* = 0.0344), regulation of autophagy (*p* = 0.0384), nucleotide excision repair (*p* = 0.0410), purine metabolism (*p* = 0.0459) and two amino acid-related metabolism pathways including glycine, serine and threonine metabolism (*p* = 0.0097), and biosynthesis of amino acids (*p* = 0.0254). The mRNAs on the stable B compartment were significantly associated with neuroactive ligand-receptor interaction (*p* = 3.96E-15), cell adhesion molecules (CAMs) (*p* = 0.0052), calcium signaling pathway (*p* = 0.0116), mucin type O-Glycan biosynthesis (*p* = 0.0252), glycosaminoglycan biosynthesis-heparan sulfate/heparin (*p* = 0.0467) and vascular smooth muscle contraction (*p* = 0.0482) (Figure 4D; Supplementary Table S4).

Given that miRNA can act as a post-transcriptional regulator to participate in various physiological processes via complementarily binding to the 3′-untranslated region (3′UTR) of its target, to comprehensively investigate the potential effects of A/B compartment switching on muscle development and IMF deposition, the targets of miRNA from A to B compartment switching region and B to A compartment switching region were analyzed, respectively. A total of 4,722 genes were obtained from two intersecting software programs, revealing that miRanda and TargetScan were potential targets of miRNAs located on the A to B compartment switching region. They were mainly clustered in regulatory and metabolic processes, otherwise directly related terms to lipid metabolisms such as acyl-CoA dehydrogenase activity, coenzyme A biosynthetic process lipid homeostasis, and fatty-acyl-CoA binding (Figure 4E; Supplementary Table S5). Moreover, 1,479 potential targets of miRNAs located on the A to B compartment switching region were predicted and clustered in cellular activity, mRNA processing, and organelle-related terms (Figure 4F; Supplementary Table S5). The signaling pathway enrichment analysis indicated that the potential targets of miRNAs were significantly associated with fatty acid metabolism (*p* = 0.0029), protein processing in the endoplasmic reticulum (*p* = 0.0092), cell cycle (*p* = 0.0109), biosynthesis of unsaturated fatty acids (*p* = 0.0162), fatty acid elongation (*p* = 0.0162), glutathione metabolism (*p* = 0.0173), valine, leucine, and isoleucine degradation (*p* = 0.0352), pyrimidine metabolism (*p* = 0.0497) on the A to B compartment switching region, as well as endocytosis (*p* = 2.74E-05), apoptosis (*p* = 3.63E-05), protein processing in the endoplasmic reticulum (*p* = 0.0008), cell cycle (*p* = 0.0048), FoxO signaling pathway (*p* = 0.0115), influenza A (*p* = 0.0406) on the B to A compartment switching region (Figure 4G; Supplementary Table S5).

### Topologically Associated Domains Calling and Boundary Sliding in Breast Muscle Between the Two Breeds

**FIGURE 5:**
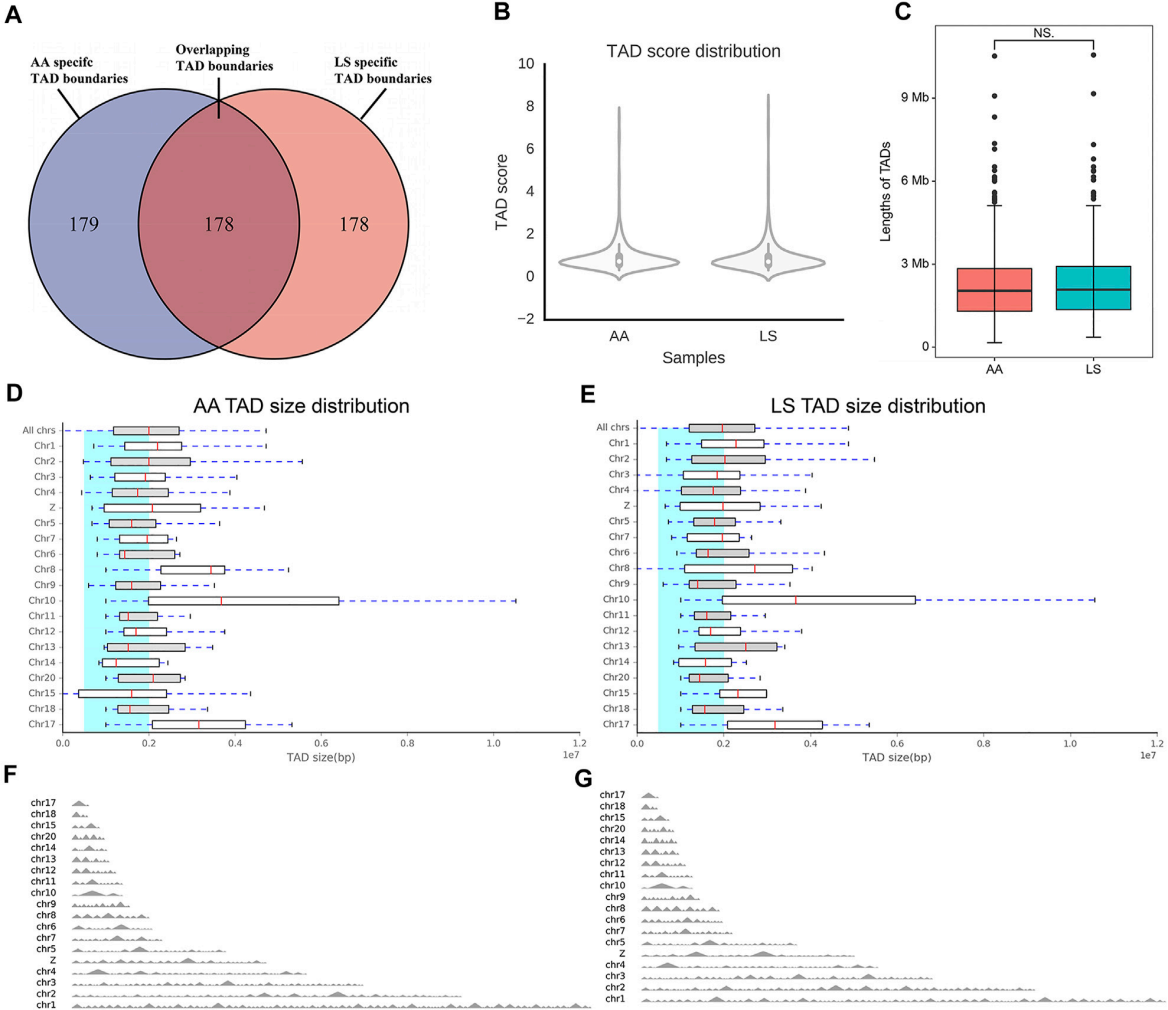
Comparative analysis of TAD between AA and LS chicken breast muscles. **(A)** Venn diagram showing that ~ 50% of all the TAD boundaries between AA and LS chicken breast muscles are conserved. **(B)** TAD score distribution of AA and LS chicken breast muscles. **(C)** Differences in the lengths of TAD between AA and LS chicken breast muscles. **(D,E)** TAD size distribution of AA and LS chicken breast muscles, respectively. **(F,G)** TAD distributions on all chromosomes of AA and LS chicken breast muscles, respectively.

The TAD boundaries were responsible for maintaining TAD independence and structural integrity, which are necessary for gene regulation. To investigate whether TAD boundaries were altered by large-scale chromosomal interactions and genomic compartment switching between AA and LS chicken breast muscle genomes, the TAD boundaries were identified by calculating the insulation plot of genome-wide interaction maps at 40 kb resolution and found that 357 and 356 TAD boundaries in AA and LS genomes, respectively. Among those, there were 178 overlapping TAD boundaries in both AA and LS, 179 AA-specific TAD boundaries, and 178 LS-specific TAD boundaries (Figure 5A). As indicated in Figure 5B, AA and LS did not show a significant change in TAD score distribution on whole chromosomes. In terms of the average size of TADs, there was no significant change between AA and LS (Figure 5C), wherein TADs on Chr10 were charactered by the largest average size in length in both AA and LS genomes (Figures 5D,E). The TAD distributions on all chromosomes of AA and LS were shown in Figures 5F,G, respectively, indicating Chr1 possessed a maximum number of TAD and Chr17 possessed a minimum in these chromosomes with TADs.

**FIGURE 6:**
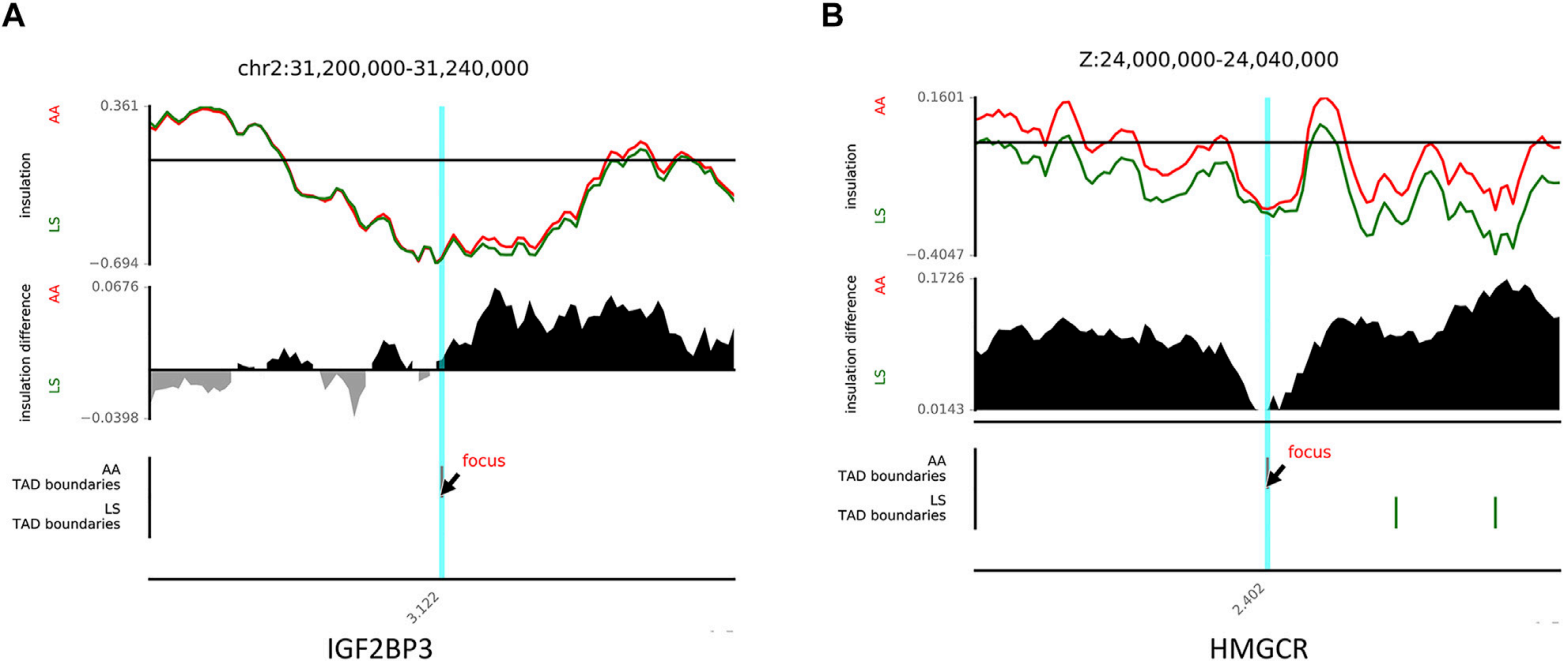
The examples of disappearing and reforming of TAD. **(A)** TAD disappearing showing an example of TAD boundary between AA (red) and LS (green) on chr2 (chr2:31,200,000-31,240,000). **(B)** TAD reforming showing an example of TAD boundary between AA (red) and LS (green) on chrZ (chrZ:24,000,000-24,040,000).

It has been well acknowledged that the dynamic changes of TAD boundaries give rise to the reforming and/or disappearing of TAD, resulting in the alternation of regulatory activity and following abnormal gene expression and subsequent disease phenotypes. There were 29 reforming events (Chr1, 2, 3, 4, 6, 7, 8, 14, 20, Z) and 32 disappearing events (Chr1, 2, 3, 4, 5, 6, 13, 15, Z) of TAD boundaries existing in AA versus LS chicken breast muscle. A total of 50 and 67 genes, were located on AA and LS TAD boundaries, respectively (Supplementary Table S6). And the 50 genes were clustered into two GO terms including defense response to the bacterium (*p* = 0.00402) and extracellular region (*p* = 0.00509). The 67 genes were clustered into 11 terms, including defense response to Gram-negative bacterium (*p* = 1.2575E-05), defense response to Gram-positive bacterium (*p* = 1.41986E-05), innate immune response (*p* = 0.00086), membrane disruption in other organisms (*p* = 0.00378), negative regulation of gene expression (*p* = 0.00672), mitochondrion (*p* = 0.00891), cytolysis (*p* = 0.02061), positive regulation of gene expression (*p* = 0.02377), lipopolysaccharide binding (*p* = 0.02779), developmental growth (*p* = 0.03351) as well as cell death (*p* = 0.03717) (Table 4). Of which, insulin like growth factor 2 mRNA binding protein 3 (IGF2BP3), a histone modification factor as m6A readers involving m6A RNA methylation were found in AA- specific TAD boundary, implying that there was probably a connection with the formation of TAD (Figure 6A). Another lipid-related gene, 3-hydroxy-3-methylglutaryl-CoA reductase (*HMGCR*) was located in the AA-TAD boundary, whereas in LS-TAD interior regions (Figure 6B).

**TABLE 4:** GO clusters of genes located on TAD boundaries of AA and LS chicken breast muscles.

Breed	Category	GO_ID	GO_Terms	*p*-value
LS	BP	GO:0050829	defense response to Gram-negative bacterium	1.2575E-05
	BP	GO:0050830	defense response to Gram-positive bacterium	1.41986E-05
	BP	GO:0045087	innate immune response	0.00086
	BP	GO:0051673	membrane disruption in other organism	0.00378
	BP	GO:0010629	negative regulation of gene expression	0.00672
	CC	GO:0005739	mitochondrion	0.00891
	BP	GO:0019835	cytolysis	0.02061
	BP	GO:0010628	positive regulation of gene expression	0.02377
	MF	GO:0001530	lipopolysaccharide binding	0.02779
	BP	GO:0048589	developmental growth	0.03351
	BP	GO:0008219	cell death	0.03717
AA	BP	GO:0042742	defense response to bacterium	0.00402
	CC	GO:0005576	extracellular region	0.00509

Note: BP, represents biological process; CC, represents cellular component; MF, represents molecular function.

### Transcriptional Expression Profiles of *IGF2BP3* and *HMGCR* in Chicken Breast Muscle Tissues

**FIGURE 7:**
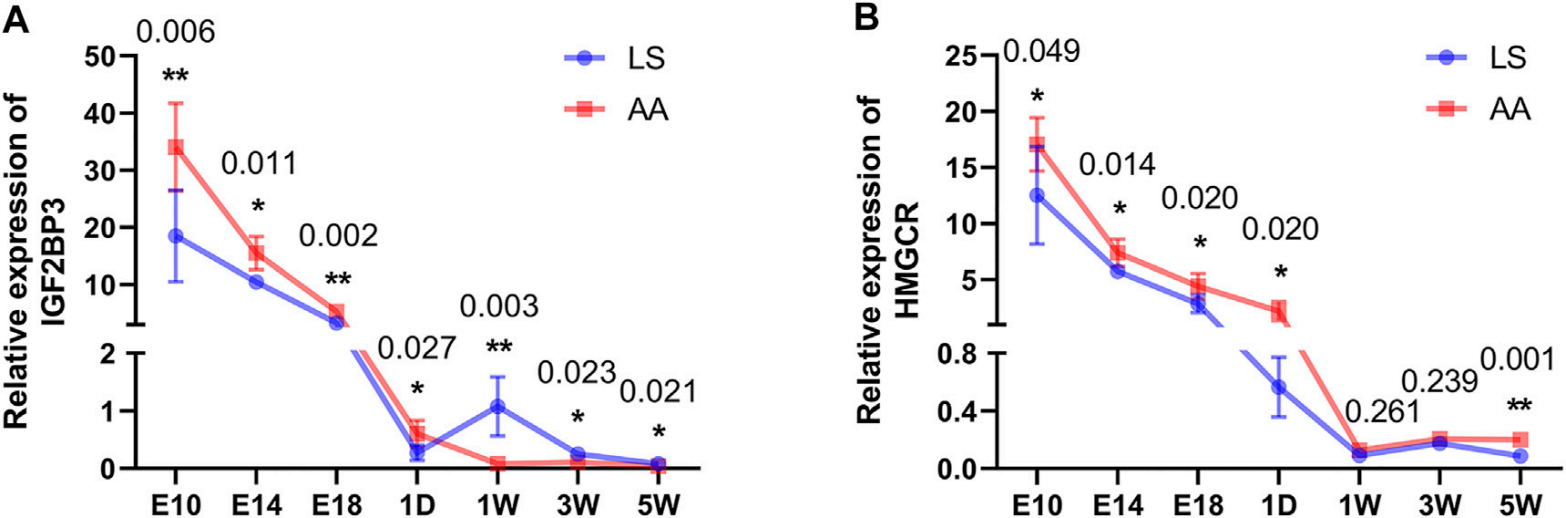
Validation of mRNA expression of *IGF2BP3* and *HMGCR* candidates in breast muscles from AA and LS chicken by qRT-PCR. **(A)** *IGF2BP3* mRNA expression in breast muscles from AA and LS chicken by qRT-PCR. **(B)** *HMGCR* mRNA expression in breast muscles from AA and LS chicken by qRT-PCR. The numbers over each comparison indicate *p* value.

To determine the difference in the mRNA abundance of the candidates, the relative expression levels of *IGF2BP3* and *HMGCR* genes in each developmental stage of chicken breast muscle were quantified by qRT-PCR. The expression of *the IGF2BP3* gene was gradually decreased as breast muscle development was processed in both AA and LS chickens. However, a significantly increased *IGF2BP3* mRNA expression was observed in the breast muscle of the AA broiler compared to LS chicken during all breast muscle development stages (*p* < 0.05) (Figure 7A). Similarly, the mRNA abundance of the *HMGCR* gene exhibited a gradually decreased trend during breast muscle development stages in LS chicken and AA broiler, while *HMGCR* mRNA expression was overall up-regulated in breast muscles of AA broiler than LS chicken, and was especially significantly higher at E10 (*p* = 0.049), E14 (*p* = 0.014), E18 (*p* = 0.020), 1D (*p* = 0.020), and 5W (*p* = 0.001) (Figure 7B).

## Discussion

Chicken has been widely accepted as a crucial research model organism for implications on phylogenetics and embryology, along with agriculture and biology, such as adipogenesis, development, immune, medicine, and cancer, owing to its convenience for operation and the extensive conservation of synteny between chicken and mammals (Burt, 2007; Beacon and Davie, 2021). Both muscle development and IMF deposition are complex and precisely orchestrated processes mediated by a network of regulatory factors directly and indirectly related to growth development and lipid metabolism. Increasing investigations are committed to elucidating these regulatory mechanisms at genomic, transcriptomic, and proteomic scales, where the genome has been deemed a linear molecular model. However, this is insufficient when it comes to revealing the connection between discrete regulatory elements, structural variations, and gene function, and consequently, it is imperative to apply three-dimensional genomics based on the spatial conformation of chromatin to explain the regulatory mechanism of gene expression. Therefore, in this study, we first identified a comparative analysis of genome-wide interactions between AA and LS chicken breast muscles upon hatching, when IMF has been proved to accumulate earliest, using Hi-C to illuminate the regulatory mechanism underlying chicken muscle development and IMF deposition at the three-dimensional chromosome scale.

Different from mammals whose lipid metabolism occurs in adipose tissue, it is the liver that principally governs lipid metabolism in chicken, where over 90% *de novo* fatty acids are synthesized. Endogenous lipids synthesized by the liver and exogenous sources absorbed from the diet undergo catalytic reaction into acyl-CoA and subsequently metabolization to form TG, phospholipids, and total cholesterol, which are incorporated into very low-density lipoprotein (VLDL) by apolipoprotein and then transported in the blood circulation (Leveille et al., 1968; O'Hea and Leveille, 1969). After that, VLDL is hydrolyzed into fatty acids by lipoprotein lipase (LPL) and then uptaken by fat cells in muscles, and reesterified to form TG, which is stored to enhance the IMF deposition (Qiu et al., 2017). Therefore, serum TG, T-CHO, and VLDL content mostly reflect lipid metabolism activity in chicken, and serum VLDL has been widely regarded to have a strong correlation with fat deposition (Griffin and Whitehead, 1982; Hermier, 1997). Our results showed the increased serum TG, T-CHO, and VLDL levels, together with enhanced accumulation of lipid droplets in breast muscle of LS chicken compared with that in breast muscle of AA broiler. It implied that more IMF deposition in LS chicken breast muscle might benefit from dramatically increased lipid metabolism in LS compared with AA, consistent with those signaling pathways related to lipid metabolism could contribute to the high capacity of IMF accumulation (Cui et al., 2012; Liu R. et al., 2017). Interestingly, LS chicken has markedly superior IMF content to AA broilers, while there was no significant difference of muscle T-CHO between them, suggesting a lower intramuscular cholesterol proportion of fat in breast muscle of LS chicken than that of AA broiler, indicating that the meat has lower cholesterol. Moreover, the accretion rate of IMF depends upon the muscle growth rate, that is, increasing muscularity could dilute the final fat content of muscle (Hocquette et al., 2010). Here, our study showed a larger breast muscle fiber diameter in AA than that in LS, to some extent, those might also explain why more IMF deposition existed in LS chicken breast muscle.

As expected, the normalized chicken Hi-C maps of AA and LS chicken breast muscle displayed a typical plaid-pattern and strong signals along diagonals, rooted in striking nonrandom patterns in its genomic sequence composition (Bickmore and van Steensel, 2013). The interactions occurred most frequently in *cis* and decay with genomic distance, in agreement with a previous report revealing that the intensity of chromatin interaction decreased as the linear distance of the genome increased, and is higher within the same chromosome than between different chromosomes (Lieberman-Aiden et al., 2009). In mammalian cell nuclei, the distribution of chromosomes is not random but closely concerned with gene density, chromosome size, GC content, and transcription activity, etc, wherein the gene-rich chromosomes concentrate at the center of the nucleus and the gene-poor chromosomes are located towards the nuclear periphery (Sun et al., 2000; Boyle et al., 2001; Parada et al., 2003; Misteli, 2007; Lieberman-Aiden et al., 2009). Our studies have revealed that, in both LS and AA breast muscle, the small gene-rich chromosomes displayed increased inter-chromosomal interaction frequency between pairs of chromosomes compared with large gene-poor chromosomes, corresponding to these chromosomes being equipped with a widely acknowledged strong physical proximity and frequent colocalization in the center of the nucleus. The formation and disappearance of chromatin interactions have been widely acknowledged to play a critical role in cell fate determination and differentiation, cooperating with gene-specific expression regulation (Bartkuhn and Renkawitz, 2008). Of note, inter-chromosomal interaction frequency showed that the large, gene-poor chromosomes in LS chicken breast muscle preferentially interacted with each other compared to AA broiler, speculating that it might contribute to explaining the difference of muscle development and IMF deposition, which needs to be further studied.

It has been acknowledged that compartment A has a more open spatial structure, higher gene density, stronger gene expression activity, and higher GC base content; on the contrary, chromatin compartment B contains centromeres and has a more closed spatial structure with lower internal gene density and lower transcriptional activity. Within the same type of compartment, chromatin interaction frequency is higher, and at the same linear distance, the interaction frequency between B type compartments is superior to A type compartments. In our study, the B compartment of either LS or AA chicken breast muscle covered approximately 60% of the chicken genome, in accordance with the point in rice (Liu C. et al., 2017). There was no significant difference in the number of B type compartments between the two breeds, more A type compartments in AA being likely to cause the dynamic regulation of gene expression related to muscle growth and development and/or lipid metabolism, resulting in a difference in muscle development and IMF deposition between AA and LS chickens. During muscle development, the programmed, controllable and orderly expression of a large number of genes leads to the specific expression of muscle cell genes, forming a complex regulatory network and signaling pathways. Simultaneously, IMF deposition is also mediated by multiple pathways and influenced by a number of genes, especially in chickens, because, unlike mammals, the liver is principally responsible for lipid metabolism in avian. According to our gene annotation analysis of mRNAs located in stable A and B compartment regions, these genes were involved in the development, enzymatic activity, transport, immune response, and other processes. The chromatin organization and stability are considered to be regulated by post-translationally histone modifications, such as methylation, acetylation, phosphorylation, and ubiquitination (Elgin and Workman, 2002; Shah, 2015). Moreover, there is a mutual dependency between chromatin organization and patterns of epigenetic marks. For example, DNA methylation is confined to the A compartment in cardiac myocytes (Nothjunge et al., 2017), unmethylated CpGs are enriched in the A compartment, and methylation levels are decreased to a greater extent in the A compartment than in the B compartment in mouse embryos (Ke et al., 2017). Moreover, both global demethylation and remethylation correlate with chromatin compartments in early embryonic development in mammals (Zhang et al., 2018), etc. Interestingly, within A compartments, there are some processes involving histone modifications, for instance, histone acetyltransferase activity, ubiquitin protein ligase activity, ubiquitin protein ligase binding, ubiquitin conjugating enzyme activity, protein dephosphorylation, ubiquitin-dependent protein catabolic process, protein autoubiquitination, ubiquinone biosynthetic process, histone H4 acetylation, suggesting that more histone modifications take place in the A compartment in chicken breast muscle. Moreover, the genes located within A compartments participate in various pathways related to energy metabolism, autophagy, fatty acid metabolism, insulin signaling pathway, and Notch signaling pathway, which have been demonstrated to be involved in muscle development and/or lipid accumulation. For example, Lactate Dehydrogenase A (*LDHA*), acyl-CoA synthetases (*ACSs*) gene family, long chain fatty acid elongases (*ELOVLs*) gene family, stearoyl-CoA desaturase (*SCD*) and histone deacetylases (*HDACs*) gene family, hydroxyacyl-CoA dehydrogenase (trifunctional protein), alpha subunit (*HADHA*), *HMGCR*, and mitogen-activated protein kinases (*MAPKs*) gene family are universal genes responsible for the development of skeletal muscle and lipid metabolism (Mashek, 2013; Li et al., 2016; Kim et al., 2017). From the late embryonic stage to hatching, a great number of genes also undergoe transcription and translation to form proteins to meet the following demands of energy at hatching. This could be the reason that the genes concerned with energy metabolism are located in the A compartment in the chicken breast muscle at hatching. The alterations of compartment types (from A to B, or from B to A) mean that changes of chromatin state (from an open active state to a closed inactive state, vice versa) directly affect the inhibition or activation of gene expression. Here, the KEGG pathways showed that the genes within A to B compartment switching regions were clustered to neuroactive ligand-receptor interaction, which has been proven to exert a role in adipocyte differentiation in human adipose-derived stem cells (Guo and Cao, 2019) and bovine muscle development (Cassar-Malek et al., 2007). Furthermore, the genes within the B to A compartment switching regions were significantly involved in the calcium signaling pathway, a highly versatile intracellular signal being capable of regulating many different processes (Berridge, 2012), as well as the TGF-β signaling pathway, which could multifunctionally regulate many cellular processes including cell growth, cell differentiation, apoptosis, morphogenesis, immune regulation, wound healing, inflammation, and cancer in both adult organisms and developing embryos (Morikawa et al., 2016). These results indicate that A/B compartment switching might yield the dynamic regulation of biological processes, leading to the difference of muscle development and IMF deposition between LS and AA chicken breast muscle. It is of note that the representative genes related to lipid metabolism, such as monoacylglycerol O-acyltransferase 1 (*MOGAT1*), acyl-CoA synthetase long chain family member 3 (*ACSL3*), lipin 1 (*LPIN1*), CCAAT/enhancer binding protein gamma (*CEBPG*), together with the representative genes related to muscle growth and development, such as insulin like growth factor 2 mRNA binding protein 1 (*IGF2BP1*), insulin like growth factor 1 (*IGF1*), myosin, and heavy chain (*MYH*) gene family were found within the A to B compartment regions. Regarding B to A compartment switching regions, representative genes including Wnt family member 16 (*WNT16*), SET and MYND domain containing 2 (*SMYD2*), BTG anti-proliferation factor 2 (*BTG2*), and PR/SET domain 6 (*PRDM6*) have been confirmed to exert vital functions in both muscle development and lipid metabolism in mammals. The A/B compartment switching might generate the changes of expression levels of these genes, thereby forming the distinct ability of muscle development and IMF deposition between LS and AA chickens. However, further research is needed to identify which are the dominant genes.

An increasing number of investigations have indicated that TAD is not only a structural unit of chromatin but also a functional unit involved in many biological processes, such as genome evolution, DNA replication, and gene regulation (Galupa and Heard, 2017; Szabo et al., 2019). The stability of the TAD structure plays a crucial role in maintaining the systematical and sequential physiological activities of the organism. The reconstruction and/or disappearance of TAD boundaries would directly lead to abnormal gene expression, consequently causing diseases such as human finger deformities (Lupiáñez et al., 2015) and fetal severe limb anomalies (Trimouille et al., 2019). We found no significant difference in TAD distribution and size occurred in LS and AA chicken breast muscles. Almost 50% of all the TAD boundaries between LS and AA chicken breast muscles were overlapped, indicating that the TAD positioning in breast muscles between the two breeds was moderately conserved, and that, their transcriptional expression regulation varied. This might partly be limited by sequencing technology and the quality of the chicken reference genome. Similar to a previous report, it is hard to identify TADs within several of the smallest chicken chromosomes, which are poorly represented in the current assembly of the chicken genome (Schmid et al., 2015). While further improvement of the chicken genome assembly emerges, the TAD analysis of these tiny chromosomes in the interphase nucleus could be supplemented. It has been verified that the TAD boundary was enriched with histone modification, transcriptional start sites, RNA polymerase II, and transcriptional factors, and could be associated with the different gene distribution and expression pattern from TAD-interior regions (Lieberman-Aiden et al., 2009; Mourad and Cuvier, 2015; Hong and Kim, 2017). Consistent with this opinion, our study also showed that TAD boundaries comprised histone modification factors and transcriptional factors, indicating a certain connection between transcription and local chromatin topology in AA and LS chicken breast muscles.

It is noteworthy that the *IGF2BP3* gene, a family of RNA-binding proteins that also acts as an RNA N6-methyladenosine reader, is located on the AA unique TAD boundaries, which were related to mRNA stability and translation. It could regulate the production, localization, and expression of downstream genes, especially IGF2, acting as a master switch governing the initiation of skeletal muscle development (Nielsen et al., 1999; Chao and D'Amore, 2008; Huang et al., 2018). A recent study demonstrated that knockdown of the *IGF2BP3* gene significantly delayed differentiation and induced proliferation of C2C12 myoblasts, and revealed that *IGF2BP3* might be considered as a candidate gene in pig breeding for meat production traits (Yang et al., 2021). Additionally, *IGF2BP3* has been identified as a potential candidate gene related to IMF deposition in chicken, and the mRNA expression of *IGF2BP3* gene in the insulin pathway was decreased in skeletal muscle of dwarf chicken, whose IMF content was significantly increased, compared with normal chicken (Lin et al., 2012; Ye et al., 2014), which is consistent with our present study that there is lower *IGF2BP3* expression in LS chicken than fast-growing AA broiler. This evidence further implies that *IGF2BP3* might play indispensable roles in breast muscle development and intramuscular fat deposition in chicken. HMGCR could drive the catalyzed conversion of HMG-CoA into mevalonic acid, a rate-limiting step in cholesterol synthesis, thus playing a critical role in cholesterol homeostasis (Sharpe and Brown, 2013). It has been reported that the polymorphisms of the *HMGCR* gene were associated with several lipid deposition- and cholesterol-related traits, and also with IMF content of *gluteus medius* muscle in pigs (Canovas et al., 2010; Chalupová et al., 2012). In bovine intramuscular adipocytes, the alteration of the *HMGCR* gene could affect the expression abundance of AMP-activated protein kinase (AMPK) and sirtuin type 1 (SIRT1), which exert important roles in the regulation of energy metabolism (Lin et al., 2020). The polymorphisms in the 3′UTR of the *HMGCR* gene exhibited significant association with the total cholesterol content in chicken muscle. This evidence shows a strong correlation between *HMGCR* gene expression with IMF deposition in livestock and poultry (Cui et al., 2010). In our present study, the decreased mRNA expression of the *HMGCR* gene was detected in the breast muscle of LS chicken compared to the AA broiler, in accordance with the lower T-CHO proportion of LS chicken, leading to the speculation that LS could be managed by a slower rate of intramuscular cholesterol synthesis. It was also evidenced that anti-HMGCR could inhibit muscle cell fusion and trigger atrophy on fully differentiated myotubes *in vitro* primary human myoblasts/myotubes isolated from human muscle biopsies of nonmyopathic patients, suggesting that HMGCR could facilitate muscle development in humans (Arouche-Delaperche et al., 2015). Additionally, RNAi-HMGCR specifically in the corpus allatum yields dwarf flies, suggesting HMGCR could control development in Drosophila (Belgacem and Martin, 2007). In chicken, polymorphism of the *HMGCR* gene was reported to correlate with leg muscle weight and leg muscle fiber diameter, revealing that the *HMGCR* gene might also exert a crucial role in muscle development (Wei et al., 2012). The lower levels of *IGF2BP3* and *HMGCR* mRNA expression during breast muscle development in LS chicken might contribute to the suppressive effect on breast muscle development and the stimulative effect on IMF deposition. Given that classical TAD-boundary regions were linked to the transcriptional control of mammalian genomes (Dixon et al., 2012), it has been proven that, compared to TAD-interior regions, the genes located in TAD-boundary regions were prone to being highly expressed (Dong et al., 2018). This might explain the higher expression profiles of *HMGCR* genes in one-day-old AA chicken breast muscles, which are located in the AA TAD-boundary regions, but in LS TAD-interior regions, as well as higher expression profiles of *IGF2BP3* gene located on the AA unique TAD boundaries. Collectively, this implies that the TAD boundary disappearing or sliding in LS chicken verse AA chicken could contribute to lower transcriptional expression of *IGF2BP3* and *HMGCR* genes in LS chicken breast muscles, and was thus responsible for slower muscle development as well as higher IMF content and lower intramuscular cholesterol proportion in LS chicken, thereby suppressing the breast muscle development but yielding excellent meat quality in LS chicken. However, the specific biological functions and regulatory mechanism at chromatin level underlying chicken breast muscle development and IMF deposition require further research.

## Conclusion

In conclusion, the chromatin structure of the chicken breast muscle from LS and AA broiler chickens was identified and compared using large-scale chromosomal *cis*- and *trans*-interactions to examine genomic compartmentalization (A/B compartments) and TAD formation. Two genes *IGF2BP3* and *HMGCR* regulated by TAD boundary sliding were identified as potential biomarkers for chicken breast muscle development and IMF deposition. To our best knowledge, our findings are the first to illuminate the regulatory mechanism of muscle development and IMF deposition at a three-dimensional chromosomal level in chickens, providing new insight into the functional role of chromatin organization in the formation of avian economic traits.

## Data Availability

The datasets presented in this study can be found in online repositories. The names of the repository/repositories and accession number(s) can be found below: https://www.ncbi.nlm.nih.gov/, PRJNA757147.
