# Supplementary material for: Chromatin Interaction Responds to Breast Muscle Development and Intramuscular Fat Deposition Between Chinese Indigenous Chicken and Fast-Growing Broiler
Source: Front Cell Dev Biol. 2021 Nov 29;9:782268. doi: 10.3389/fcell.2021.782268 (PMC8667342; doi:10.3389/fcell.2021.782268)
Supplement: Supplementary file 1 [file DataSheet1.PDF]

Supplementary Material

Supplementary Figures

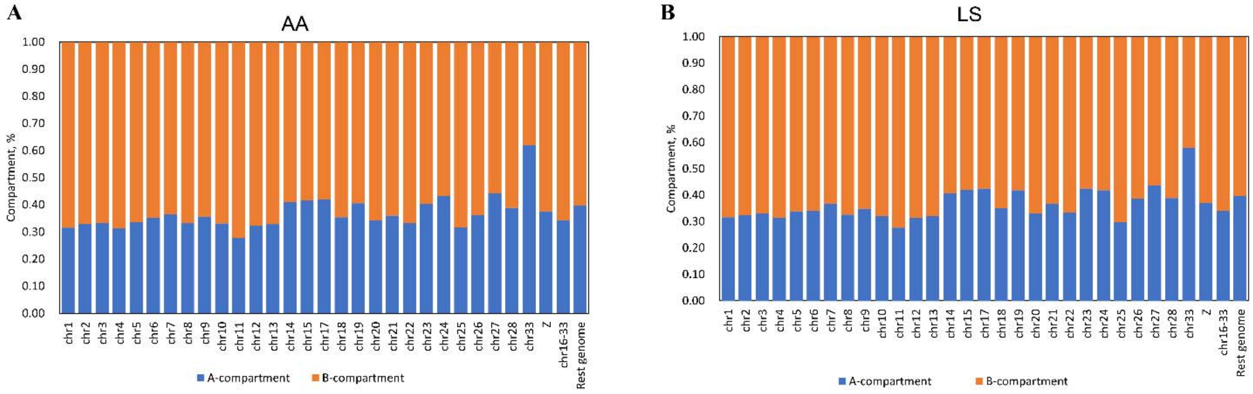

Supplementary Figure 1. The distribution proportion of A compartment and B compartment across chromosomes in AA **(A)** and LS **(B)**.

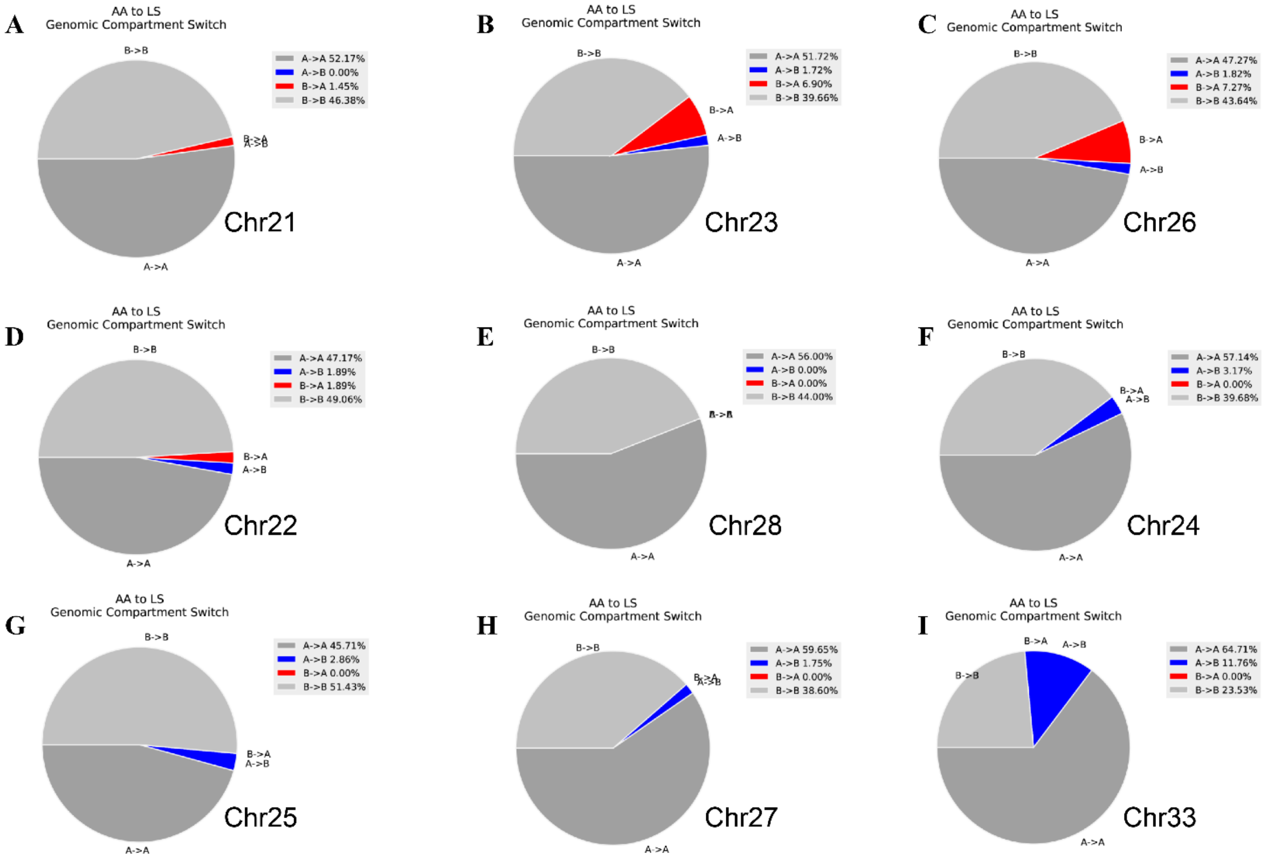

Supplementary Figure 2. The distribution percentage of A_to_A, A_to_B, B_to_A, B_to_B compartment switching in chr21 **(A)**, chr23 **(B)**, chr26 **(C)**, chr22 **(D)**, chr28 **(E)**, chr24 **(F)**, chr25 **(G)**, chr27 **(H)**, chr33 **(I)**.

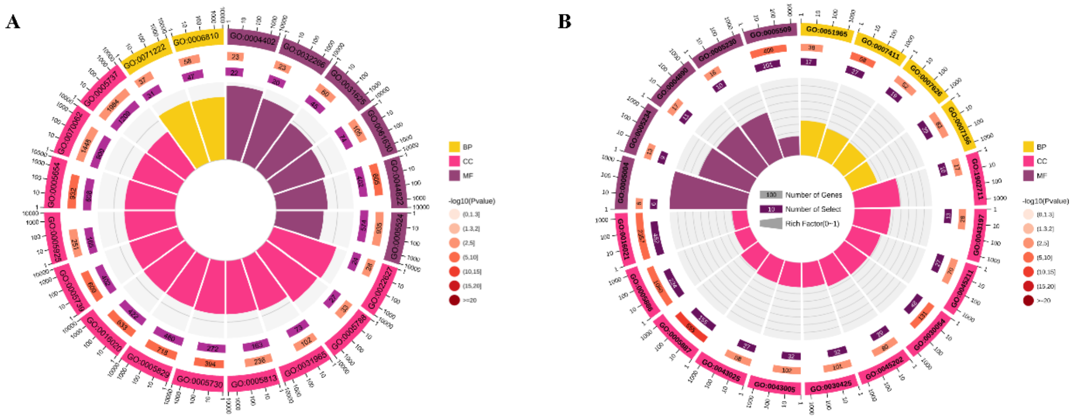

Supplementary Figure 3. GO enrichment analysis of genes located in A_to_A compartment **(A)** and B_to_B compartment **(B)**.
